# Formalising remote consulting in primary care services for underserved populations with long-term conditions in sub-Saharan Africa: a process evaluation and stakeholder engagement

**DOI:** 10.1136/bmjgh-2024-018780

**Published:** 2026-10-06

**Authors:** Olufunke Fayehun, Bronwyn Harris, Titus Mashanya, Kennedy Bashan Nkhoma, Aboluwaji Daniel Ayinmoro, Ayanfeoluwa Ibrahim, Abiodun Alliu, Wendy Higman, Chinwe Onuegbu, Akinyinka Omigbodun, Senga Pemba, Jackie Sturt, Frances Griffiths, the REaCH Trials Collaborative Group, Jackie Sturt

**Affiliations:** 1 Sociology, University of Ibadan, Ibadan, Nigeria; 2 Warwick Centre for Global Health, Warwick Medical School, University of Warwick, Coventry, UK; 3 School of Medicine, St Francis University College of Health and Allied Sciences, Ifakara, Tanzania, United Republic of; 4 Florence Nightingale Faculty of Nursing Midwifery and Palliative Care, King’s College London, London, UK; 5 Cicely Saunders Institute of Palliative Care and Rehabilitation, London, UK; 6 School of Psychology, Social Work and Public Health, Oxford Brookes University, Oxford, UK; 7 University of Ibadan College of Medicine, Ibadan, Nigeria; 8 Centre for Health Policy, University of the Witwatersrand, Johannesburg-Braamfontein, South Africa

**Keywords:** Health Services Accessibility, Health policy, Delivery of Health Care, Health systems, Universal Health Care

## Abstract

**Introduction:**

Remote consulting can improve healthcare access and equity. When delivered by trained health workers, it is safe, trustworthy and efficient. Alongside two independent clinical trials, we studied the implementation of a cascaded health worker training intervention (REaCH), plus airtime, on remote consulting in primary healthcare facilities in Nigeria (n=35) and Tanzania (n=21). Additionally, we assessed health service readiness for sustainable remote consulting in Ghana, Kenya, Nigeria, Malawi, Sierra Leone, Tanzania and Uganda.

**Methods:**

We explored what worked for whom, how, and why in both health worker training in Nigeria (n=115) and Tanzania (n=136), and the implementation of remote consulting for patients with long-term conditions. We assessed REaCH training for talent, resources, alignment, implementation and nurture. We analysed system readiness with WHO’s Digital Investment Guide. We undertook 82 semi-structured interviews with health workers and patients (n=40: Nigeria, n=42: Tanzania), collected text produced, engaged with stakeholders and reviewed key policies/guidelines. Data were analysed thematically.

**Results:**

Health workers were already doing informal remote consulting. REaCH training helped facilities to plan and implement remote consulting. It addressed concerns about delivering a safe and ethical service. Provision of airtime was critical. Health workers used remote consulting for patient follow-up and patients sought advice, test results, prescriptions, referrals and emergency help. Patients and health workers trusted and appreciated remote consulting as it saved time. Patients felt cared for, but some received calls at inconvenient times. Health workers reported reduced stress but noted off-duty calls could be burdensome. Stakeholders were aware of informal remote consulting and were concerned about quality. None of the study countries provided airtime for remote consulting or had guidance or recording systems for remote consulting.

**Conclusion:**

There is an appetite for remote consulting in primary healthcare. However, health systems in seven countries of sub-Saharan Africa are not yet fully prepared for this.

**WHAT IS ALREADY KNOWN ON THIS TOPIC**

- Informal remote consulting is widespread in sub-Saharan Africa, with risks for patient safety, confidentiality and equitable healthcare access. In primary care, our clinical trials indicate that health workers who are trained in how to provide remote consulting for their patients with long-term conditions (REaCH) and given airtime and a facility mobile phone to do this, deliver a safe and trusted service embedded in their routine work.

**WHAT THIS STUDY ADDS**

- REaCH training aligns with the learning needs of health workers, enables them to integrate remote consulting into their routine work and allays ethical and safety concerns about remote consulting. Patients appreciate the option of remote consulting. Health systems are not yet ready for scale-up.

**HOW THIS STUDY MIGHT AFFECT RESEARCH, PRACTICE OR POLICY**

- For scale-up of remote consulting in countries of sub-Saharan Africa, there is need to recognise remote consulting in health workers’ scope of practice and as a formalised, normalised activity within health facilities, to deploy service delivery guidelines and secure funding for airtime for healthcare facilities.

## Introduction

 In sub-Saharan Africa, telemedicine has a long history of use to improve access to healthcare and provide healthcare education.^1^ It is a strategy for strengthening digital health systems and delivering universal health coverage on the continent.^2 3^ Telemedicine is viewed as a means for creating a pan-African Digital Single Market, with a connected health service and workforce.^4^ Its implementation has been accelerated through technological change and the COVID-19 pandemic.^2^ However, in many low-resource contexts, barriers impede its implementation and scale-up. These include lack of funding, weak infrastructure, data insecurity and privacy challenges, shortages of skilled workers and competing health system priorities.^4 5^ The African Union’s Digital Transformation Strategy (2020–2030) recommends that member states adopt policies for teleconsultations and e-prescriptions to facilitate their ‘smooth integration with current clinical practices’ and to address ethical concerns (p.34).^4^ The WHO advocates using telemedicine to complement face-to-face services (not replace them) and the establishment of monitoring systems and standard operating procedures for patient consent, data security, provider licensing and accreditation, and accountability.^2 6^

In this paper we focus on one patient-facing form of telemedicine, namely remote consulting.^7^ Remote consulting involves a person with a perceived health need consulting a healthcare provider using a mobile phone (their own or borrowed), using the telecommunications infrastructure or internet.^8^ It is a relatively simple innovation, requiring only that consulting parties have access to a charged mobile phone connected to a mobile network.

Informal remote consulting, where health workers use their personal phones for delivering healthcare often at their own financial cost, is widespread in sub-Saharan Africa.^9^ Nurses, doctors and community health workers report using their own phones extensively in their work.^10–12^ This brings ethical risks, including unrecorded contact between health worker and patient, invasion of health worker privacy and potential confidentiality breaches.^13^ Because informal remote consulting relies on social networks and personal contacts, it may also be exclusionary. Bringing remote consulting formally into the routine provision of healthcare is a first step towards mitigating these risks and valuing remote contact between patients and health workers,^14^ while also improving health equity. Alongside informal consulting practices (usually at local community level), national or regional remote consulting services run by commercial companies, government agencies and/or non-governmental organisations, are common across sub-Saharan Africa, often operating independently of local primary care facilities, with patients contacting them directly.^12^ In contrast to informal and national/regional provision, in this paper, we describe the process of training health workers to plan for formal remote consulting as part of their healthcare practice in local primary care facilities. We explore how health workers planned and implemented remote consulting in their own facilities, and health system challenges for scaling-up formal remote consulting in primary care in sub-Saharan Africa.

### Cluster trials alongside this study

Our study ran alongside two independent cluster step-wedge trials in Nigeria and Tanzania (2021–2022). These aimed to assess the impact of a health worker training intervention (REaCH)—on rates of remote and face-to-face consultations, safety, trustworthiness and implementation feasibility^15^ (box 1). Trial findings showed that in Nigeria, remote consultations increased fourfold without a decrease in face-to-face consultations. In Tanzania, face-to-face and remote consulting rates were unchanged. Prescribing rates, investigation rates and trustworthiness scores showed no difference—the status quo was maintained, regardless of consultation mode. Training was accessed by six cadres of health workers of both genders, either directly or through training cascade. Engagement with the training was high and the pass rate was >70%. The scores for acceptability, appropriateness and feasibility were high. The trials indicated that REaCH training can be implemented and that the remote consulting undertaken by the trained health workers is safe and trustworthy.^15^

Box 1 **The REaCH training intervention: a process for planning and doing remote consulting at facility-level**

REaCH training is designed to increase the use and quality of remote consulting. It targets health workers of any cadre who are engaged in delivery of primary care or other ambulatory services. Registered health workers (Tier 1 trainees) at facility level are trained to practise remote consulting, to plan, implement and support remote consulting in their facility, and to pass on their learning to other cadres of workers (Tier 2 trainees). The training does not teach clinical skills. It provides a framework for health workers to think through access to remote consultation for facility patients, how to deploy it and how to mitigate inequalities. The curriculum comprises eight modules including adaptation of communication in consultations when remote from the patient, how to plan for remote consulting in their health facility, use of behaviour-change techniques to achieve change, leadership and cascading of the training to other cadres (online supplemental materials 1 and REaCH training for health workers in Remote Consulting).

The facilitated Tier 1 training is delivered remotely and involves self-directed learning with peer-support and facilitation. REaCH training was piloted with 63 health workers in Ifakara, Tanzania and adjustments made.^22^ In the REaCH trials,^15^ the intervention comprised three interacting components:

1. Tier 1 trainees undertook facilitated learning delivered remotely through Moodle, a smartphone app (22 hours), plus, where available, additional WhatsApp engagement with peers and a local REaCH facilitator (employed in our team) over 14 days using the health worker’s smartphone. Tier 1 trainees were doctors, nurses and medical officers who worked for primary healthcare facilities. Facilitators were experienced clinical teachers.
2. Cascaded Tier 2 training was delivered by Tier 1 trainees to other health workers in their facility (Tier 2 trainees) in English and/or local languages as appropriate. Cascaded training consisted of 4 hours of learning per day delivered face-to-face by the Tier 1 trainees (now the trainers) and, where available, additional WhatsApp engagement—over 4–5 days. Tier 1 trainees purposively chose which health workers to include in the cascade.
3. Provision of airtime/data allowance for Tier 1 and 2 trainees to support training participation and delivery of remote consulting.

To facilitate the transfer of project data, each participating facility was provided with one smartphone.

Our approach was to provide frontline health workers with knowledge, skills and tools to help them work out *in their own context* how to deliver remote consulting to their own patients living with long-term conditions, as part of their normal care delivery.

### Study aim and objectives

This study aimed to evaluate REaCH training and implementation and to explore policy readiness for the scale-up of remote consulting in primary care within sub-Saharan Africa.

Our research in Nigeria and Tanzania sought to:

1. Evaluate the delivery of REaCH training and its cascade.
2. Understand how health facilities operationalised the training they had received in their daily practice, how remote consulting was experienced by the health workers and their patients and how challenges were met.

Our stakeholder engagement sought to:

1. Identify gaps in policy and guidance that would need to be filled to enable implementation of remote consulting in primary healthcare for people living with long-term conditions.

## Methods

### Study design and conceptual frameworks

Our study was guided by the UK’s updated Medical Research Council framework for developing and evaluating complex interventions, which recognises the importance of context, systems, stakeholders and processes in bringing about health system change.^16^ During our data collection and analysis, we explored what works (or does not work) in training, planning and implementing remote consulting, for whom, how and why. We sought to identify uncertainties, local adaptations and workarounds to support ‘real-world decision-making’ towards scale-up.^16^ Fidelity and quality of implementation were reported with the cluster trial results.^15^ Here, we evaluate the intervention using the Talent, Resources, Alignment, Implementation and Nurture (TRAIN) framework^17^ to assess talent, resources, alignment, implementation and nurture in the REaCH training process. Mormina and Pinder suggest that the sustainability of a cascaded training approach depends on these ‘constitutive elements’ (np.) being present or developing at different structural levels: individually (facilitators and trainees), organisationally (REaCH trials and participating primary healthcare facilities) and supra-organisationally (national health and technology systems, Nigeria and Tanzania).^17^

### Study setting and population

Our research in Nigeria and Tanzania was undertaken in the same study sites as the REaCH trials (table 1)^15^: 35 government-run primary care facilities in urban/peri-urban Nigeria; and 21 primary care facilities in rural Tanzania (a mix of state-funded, non-governmental organisation-funded and private-for-profit facilities). The facilities were grouped into 20 clusters for each trial. Participating health facilities created a register of patients aged 18 or older, who had consulted at least three times over the previous year for one of four long-term health conditions—hypertension, diabetes, coronary heart disease and chronic obstructive airways disease. These patients formed the trial open cohorts of 8776 (Nigeria) and 3246 (Tanzania) patients. Facility managers nominated trainees in line with their facility’s gender profile.

**Table 1:** Study setting and data sources from the REaCH training trials in Nigeria and Tanzania

Context/data source				Nigeria	Tanzania
Study location and health system	**Health systems in both countries** Are three-tiered (tertiary, secondary, primary) PHC (study focus) is largely decentralised to local government authorities (Nigeria) or district councils (Tanzania). PHC systems comprise: Small health posts and clinics (Nigeria) or dispensaries (Tanzania), which provide basic preventative and curative services. Larger health centres that offer inpatient and higher-level services. Referrals are made to district (Tanzania) and secondary-level general (Nigeria) hospitals. Health systems are pluralistic: with a mix of public and private sectors (for-profit and not-for-profit). The REaCH trials included all sectors in Tanzania and only public sector facilities in Nigeria. In both countries, all facilities sampled for the five in-depth clusters were public sector health centres.			**Ibadan Metropolis, Oyo State:** highly populated urban centre, with peri-urban and remote rural communities.	**Morogoro Region:** mostly remote rural areas with small villages, some urban centres and peri-urban areas with small towns.
				Facility phone numbers are publicly available. Consultations are (mostly) free at point of use. Medications/tests are usually paid out-of-pocket, to facility of patient’s choice. Where a patient is known to clinic staff, prescriptions can usually be collected for them.	Facility and doctor phone numbers are publicly available. User fees (with some exemptions). Fees are paid in person at health facilities (there is no electronic payment system). Patients must collect medication from facilities in person. Due to public sector drug stock-outs, people often pay out of pocket to private pharmacies for their medication.
All REaCH trial clusters (n=20)	Moodle app	Number of Tier 1 trainees enrolled to use the app		20	42
		Tier 1 trainees who participated in the Moodle Student Forum (written text)		0/20	25/39
	WhatsApp training chats	Tier 1 WhatsApp training chats (out of total facilitated trainings) (text and voice recordings)		9/9	7/7
		Tier 2 WhatsApp training chats (out of total cascaded trainings) (text and voice recordings)		20/20	11/20
	Cascade report	From Tier 1 trainer (out of total Tier 2 trainings cascaded) (written text)		14/20	20/20
In-depth clusters (n=5)		All facilities in the 5 in-depth clusters were public sector health centres		8 facilities serving residents in urban and peri-urban areas	5 facilities serving residents in rural villages and small towns
		Health worker numbers (all cadres) in the in-depth facilities (range)		4–11	24–33
		Number of enrolled REaCH patients (long-term conditions) (range across the clusters)		43–365	103–249
		Semi-structured interviews (n=women)*	REaCH facilitators	2 (1)	2 (0)
			Health facility managers†	8 (8)	5 (0)
			Tier 1 trainees	2 (0)	5 (2)
			Tier 2 trainees	10 (7)	10 (3)
			Patients	18 (12)‡	20 (13)§
			Total interviews	40 (28)	42 (20)

* Consistent with facility gender profiles, most participating health workers were women in Nigeria (16/22) and men in Tanzania (17/22). In both countries, over half of the patient participants were women (12/18, Nigeria and 13/20, Tanzania).

† Interviewed facility managers had been trained as Tier 1 trainees. In Tanzania, three trainees were unable to complete the training.

‡ In Nigeria, most patients (13/18) were self-employed traders or involved in a business. One was an engineer and another reared cattle. 16 were married and 2 widowed.

§ In Tanzania, three patients were pastoralists. 10 patients were either active (n=3) or retired (n=7) civil servants. Most had attended primary school (14/20) and two held a higher education qualification.

PHC, primary healthcare.

### Data collection

#### Training documents and reports

For the 20 clusters in Tanzania and Nigeria, we collected in digital form any written or spoken text produced during Tier 1 training on the Moodle app and WhatsApp groups. WhatsApp chats were exported to Word documents. WhatsApp text and voice-recorded messages were translated into English, transcribed and identifiers removed. For Tier 2 training, brief process cascade reports (what happened, who was involved) were written by Tier 1 trainees in English (Tanzania) or delivered verbally to a research assistant, who translated and transcribed the report (Nigeria).

Before and after each REaCH training, facilitators confirmed consent to the use of trainees’ text for analysis, clarifying that all personal identifiers would be removed before sharing with the research team. Trainees were advised that they could confidentially inform facilitators if they did not wish their text to be included.

#### Qualitative semi-structured interviews

Seeking in-depth data richness, we randomly sampled five clusters trained during the first 6 months in each country, where we undertook face-to-face interviews. In both countries, the included facilities were public sector health centres. Interviews were scheduled 7 months after the training of Tier 1 participants to give the REaCH intervention time to embed within the facility. We interviewed:

1. REaCH facilitators, facility managers and healthcare workers who had received REaCH training (as both Tier 1 and Tier 2 trainees) about the experience of the training, support and cascade process. We explored how facilities and trainees planned and deployed remote consulting, what worked (or not) in both the training and implementation of remote consulting and how they met challenges to embedding cascaded training and remote consulting at facility-level (Topic guides, online supplemental materials 2).
2. Patient trial participants about their experience of remote consulting and its impact on their healthcare, including what worked (or not) and why, expenditure on airtime and changes needed or made to enable remote consulting (Topic guide, online supplemental materials 2).

Participants were a random sample of health workers and patients drawn from those who had consented during the trials to take part in qualitative interviews. Written consent was taken before each interview. Interviews were translated into English, transcribed and pseudonymised. Table 1 summarises the study setting and data sources from the REaCH training trials used to support objectives 1–2 of our study.

#### Stakeholder engagement

To identify policy/guidance gaps affecting the implementation of remote consulting in primary healthcare for people living with long-term conditions (objective 3), we reviewed policy documents and engaged with key stakeholders in Ghana, Kenya, Malawi, Nigeria, Sierra Leone, Tanzania and Uganda.

Between February and June 2023, we reviewed online policy documents in the seven countries to assess policies related to remote consulting. We used Digital Square 2021 COVID-19 Map and Match resource country briefs^18^ (not available for Nigeria) to examine the extent of digital health innovation, including remote consulting. We extracted World Bank data on mobile cellular subscriptions to understand likely mobile phone population coverage.^19^

In 2020, in Nigeria and Tanzania, we held three workshops involving 61 decision-makers, health workers and community members^12^ to discuss remote consulting and its potential. In 2023, we engaged 89 stakeholders in Sierra Leone, Ghana, Kenya, Uganda and Malawi, as individuals and groups (range 3–38/country). Stakeholders included government policymakers, leaders of professional bodies such as Nurse and Midwifery Councils and leaders of healthcare provider organisations. We presented the findings of our cluster trials and discussed the next steps for deploying remote consulting in primary care. Meeting notes were compiled for analysis. Using the WHO Digital Investment Guide framework^6^ on the Information and Communication Technology (ICT) environment and the enabling environment, we analysed all documents for what is needed in each country to enable the embedding of local facility-based remote consulting. We identified evidence of each component and missing elements. We verified the accuracy of the information for Nigeria and Tanzania in 2023.

### Data management and analysis

All data were electronically transferred from local secure storage to a secure storage area hosted by the Clinical Trial Unit at the College of Medicine, University of Ibadan. Research teams from Nigeria, Tanzania and the UK had access to the data for data entry, quality monitoring and analysis. Data were analysed thematically.^20^ In each country team, researchers (CO, ADA) read and broadly coded the interview transcripts using NVivo (Nigeria) and Word (Tanzania). Theme codes were framed around the training process, individual and organisational changes, examples of actual remote consultations and unexpected consequences, and new themes identified (coding guide, online supplemental materials 2). A researcher (WH) read and summarised the WhatsApp chats and cascade reports for both countries. BH, OF, TM and FG read the coded data and summaries. BH extracted and summarised data relevant to the TRAIN framework. FG and BH compared data across clusters and countries and further coded the data thematically. The research team discussed themes and findings throughout the process. Analysis continued until we reached information power, with the data providing ‘sufficient basis’ (p.24) for our analysis (after Malterud *et al*, 2016 in ^20^). We used the Standards for Quality Improvement Reporting Excellence (SQUIRE) guidelines when reporting our research.^21^

### Community engagement and involvement

Prior to the REaCH trials, we consulted with local and national decision-makers, health facility managers, frontline health workers and community leaders and representatives at each site, to identify experiences, perceptions, anticipated challenges and key priorities.^12 22^ We piloted the REaCH training with 63 health workers in Ifakara, Tanzania and made adjustments.^22^ During the REaCH trials, frontline healthcare workers and facility managers received REaCH training. Patients from the study communities were enrolled in the trials.

## Results

### Evaluating the delivery of REaCH training

Table 2 outlines the health workers trained by cadre/gender and facility staff numbers. In Nigeria, REaCH training was delivered to 115 health workers (20 Tier 1 and 95 Tier 2), reaching 60% of all staff within the participating facilities. Nearly all trainees were women (n=101). In Tanzania, 136 health workers were trained (40% women), representing 13% of all staff in the study clusters when including those with technical and administrative roles, or 20% excluding this cadre. Cadres trained included 39 doctors (n=10 Nigeria, n=29 Tanzania), 68 nurses (n=30 Nigeria, n=38 Tanzania) and, in Nigeria, 75 community-based health officers and extension workers. In Tanzania, a quarter of the Tier 2 trainees (n=27) were staff with technical and administrative roles (in pharmacy, laboratory, reception).

**Table 2:** Health workers trained by cadre/gender and staff numbers (with total staff by cadre in the five in-depth clusters and all clusters)

Training (n=20 clusters)	Nigeria				Tanzania			
Cadre (women) trained*	Total trainees		Total staff by cadre		Total trainees		Total staff by cadre	
	Tier 1	Tier 2	5 clusters	All sites	Tier 1	Tier 2	5 clusters	All sites
Medical doctor	10 (2)	–	1	14	24 (8)	5 (0)	12	66
Clinical officer	–	–			11 (3)	21 (8)	19	84
Assistant medical doctor	–	–			7 (2)	6 (1)	3	46
Nurse	10 (10)	20 (20)	15	51	–	38 (17)	56	431
Community health officer	–	33 (31)	19	50	–	–	–	–
Community health extension worker	–	42 (38)	30	79	–	–	–	–
Technical and administrative staff†	–	–				27 (15)	42	372
Total trainees	20 (12)	95 (89)	65	194	42* (13)	97 (41)	144	1051

* Includes those who started the training but did not complete. In Tanzania, three of the Tier 1 trainees did not complete the training.

† Includes technicians, (radiography, pharmacy, laboratory), medical attendants and receptionist; trainees were identified by facility managers as people who assist with the delivery of healthcare to people with long-term conditions.

We evaluated the REaCH training process for TRAIN,^17^ considering each training element across three levels: individual (REaCH facilitators, Tier 1 and Tier 2 trainees), organisational (primary healthcare facilities in the REaCH trials) and supra-organisational (national health and technology systems).

Successful delivery of REaCH training was enabled by expert facilitators and engaged facility managers (*Talent*), who supported and encouraged Tier 1 participants during their training and into the cascading process. The provision of technological support by the REaCH information technology (IT) team and, importantly, airtime, were identified as crucial training enablers (*Resources*). WhatsApp provided a forum for Tier 1 participants to raise issues, seek clarification and support each other. While some WhatsApp groups were highly engaged, others were less active, particularly in Tanzania, where connectivity and access to WhatsApp itself were also more challenging. Where used, trainees mentioned that WhatsApp text and voice notes created an accessible, portable training record for easy future reference.

In both countries, health workers recognised that they had, to some degree, been practising remote consulting prior to the REaCH training but informally: ‘the structure was not there’ (Health worker, Nigeria). At an individual level, most felt that the training prepared them to better integrate remote consulting into their routine work and that it addressed their concerns about delivering a safe, ethical and professional service (*Alignment*). All Tier 1 trainees successfully cascaded the training to health workers within their own facilities (*Implementation*), having gained confidence with the training and finding organisational support in their facilities. Trainees from both tiers emphasised the importance of flexibility in the training process. They valued adaptations made to fit local technology, staffing and work routines.

Alongside content, Tier 1 trainees said they appreciated the democratic, inclusive approach modelled by the REaCH facilitators and sought to cascade this ethos in their own training (*Nurture*). In both countries, synchronous WhatsApp chats were scheduled for evenings, which most trainees found convenient. However, two women (one per country) said this caused disruptions to domestic duties. At a broader, supra-organisational level, the REaCH trials enabled real-time lesson-sharing between the training teams in Tanzania and Nigeria. South-South knowledge transfer and learning (*Resources, Nurture*) were further extended through the project team’s engagements with decision-makers and frontline staff from the seven participating countries.

Although categorised separately, there are overlaps between the TRAIN elements and across individual, organisational and supra-organisational levels. For example, individual *talent* is indicated by the enthusiasm and willingness of managers and staff to participate in the training. Simultaneously, organisational *resources* are to be found in formal facility-level agreements allocating time and space for REaCH training. Additionally, REaCH-provided airtime, IT support and facilitation served as *resources* and *nurture*. Here, the organisational level took on particular importance in the absence of supra-organisational *alignment* and *resources* for professional accreditation and formal recognition of remote consulting within health workers’ scope of practice; a wider context which raised worries about sustainability of remote consulting for several trainees in both tiers. Table 3 (online supplemental materials) brings together the TRAIN elements/levels^17^ into a more detailed overview.

**Table 3:** Health system enablers to support embedding of remote consulting within state-provided primary healthcare systems and whether they were in place (✓) or not (X) in seven sub-Saharan Africa countries in 2023

Health system building block^*^	Enabler	Ghana	Kenya	Malawi	Nigeria	Sierra Leone	Tanzania	Uganda
Leadership and governance	Legislation and policy (national): eHealth and digital health	✓	✓	✓	✓	✓	✓	✓
	Digital health programme/body (national)	✓	✓	✓	✓	✓	✓	✓
	Strategy/policy (national): telemedicine and telehealth	✓	✓	✓	✓	✓	✓	✓
	Digital health tools in use	✓	✓	✓	✓	✓	✓	✓
Medical products, vaccines and technologies†	Availability of ePrescriptions‡	✓	✓	X	✓	X	X	X
Health workforce	Remote consulting included in scope of practice of healthcare workers	X	X	X	X	X	X	X
	Accredited training available in remote consulting§	X	X	X	✓	X	✓	X
	Platform available for remote delivery of remote consulting training	✓	X	X	X	X	✓	✓
Service delivery	National agreements with telecommunication companies for provision of affordable airtime within government-funded primary care¶**	X	X	X	X	X	X	X
	Service delivery guidelines on remote consulting	X	X	X	X	X	X	X
Financing	Mechanism for payment/reimbursement of remote consulting services (eg, billable item in national health insurance scheme)	X	X	X	X	X	X	X

* Adapted from WHO Digital Investment Guide. We have excluded the Health Information Systems Building Block because we are focusing on embedding remote consulting into the existing system. In our REaCH training, we emphasise to healthcare workers the need to follow the same procedures and practices as they would when working face-to-face, for example, to use a patient’s clinical record as it would be used when consulting in-person and to follow the same procedure for making a referral.

† See findings text for ICT infrastructure, including mobile phone network subscriptions and need for a stable power supply (for connectivity and charging of devices).

‡ e-Prescriptions at least at start of scale-up.

§ Platforms in Nigeria and Tanzania have been established to host REaCH training.

¶ This does not include national remote consulting systems, for example, set up in response to epidemic.

** Local agreements are in place between NGO healthcare provider organisations and telecommunication companies for affordable airtime. These NGO provider organisations may be providing healthcare on behalf of the government.

eHealth, electronic health; ICT, Information and Communication Technology; NGO, non-governmental organisation.

### Impact of REaCH training on primary care staff and facilities

#### Planning formal, embedded remote consulting

As part of their training and in preparation for implementing remote consulting, trainees identified key considerations for effectively embedding remote consulting at facility level. These included selecting appropriate patients to undertake remote consultations, supporting patients to use technology (eg, patients with reading or hearing difficulties) and educating community members on the benefits of remote consulting. Trainees described needing to select the right mode of delivery for their patients, such as opting for phone-based consulting in communities where WhatsApp was not widely used. They emphasised that health workers must be equipped with airtime and official work mobile phones and identified the value of using behaviour change techniques (covered in the training) to motivate staff. Designating specific spaces within the facility for conducting remote consultations was also seen as key to formalising the process, along with ‘registering the names of our patients and scheduling their appointment dates’ (health worker, Tanzania).

### Implementing remote consulting

Following REaCH training, remote consulting was implemented in all participating trial facilities. Implementation varied across facilities but broadly, remote consulting was used proactively, for tasks such as following up with patients who had previously had a face-to-face visit or providing health education; and reactively, when patients called health workers seeking advice for a problem or requesting a prescription or referral (box 2).

Box 2 **Implementing remote consulting**

**Proactive use of remote consulting**

To follow-up with patients

- The patient had a cough so after attending to her [at the facility] I told her that I would call her…to know how she was feeling… so I called her then she said she has been taking her drugs as prescribed and that the cough has reduced… I called her three to four times. (health worker, Nigeria)
- When the doctor called me last time, he directed me again on how to take my pills, that is, he told me I was supposed to take two pills per day, one in the morning and the other one at night, and he then told me after two weeks I was to go back to the health centre for some laboratory tests, where the results showed that my blood sugar was going back to normal from being so high. (patient, Tanzania)

To provide health education

- We can counsel on phone…give advice to patient…malaria … they need to sleep under the mosquito net, the reason why everywhere needs to be fumigated in case there is mosquito - I did all that on phone. (health worker, Nigeria)
- …My blood sugar… I am thankful for all the explanations I get through the phone, it has strengthened my health status, I do well. (patient, Tanzania).

**Reactive use of remote consulting**

Patients phone to sort out their confusion

- After being diagnosed with high blood pressure, I was prescribed three kinds of drugs to stabilize the condition. While home, I happened to mix up the medical bottles after ingesting some tablets… I decided to make a call to consult my doctor about it to avoid taking them wrongly. When a doctor answered the call, he asked how it happened and I took him through of what happened exactly, after listening to my explanation he told me to describe the colour and the size of each kind of the drugs tablets mixed. Though I described them, the doctor could not understand well thus he instructed me to go to the nearby pharmacy with all drugs mixed so that he can get more details of them while conversing with the pharmacist. I did as I was told then everything went well. (patient, Tanzania)

Patients ask about test results

- The patient made a call (to me) and explained the number of tests he had done, in that explanation I realized that he needs to reduce the dose intake thus I told him my advice and then the case was solved. (health worker, Tanzania)

Patients ask for prescription or referral

- Some patients will just call you to give them prescriptions or to get a referral. (health worker, Nigeria)

Patients phone in an emergency

- …10 pm and I was not on duty but [my patient called with an emergency. I consulted an on-duty doctor and referred the patient to the hospital.] The doctor received him and administered treatment… an hour later, I was able to talk to both the doctor and patient about his progress. (health worker, Tanzania)

Patients ask for advice about treatments

- I spoke to [the doctor] yesterday because I received a text message about a COVID-19 vaccination… my immunity isn’t strong… So calling the doctor helped me get information. (patient, Tanzania)
- I said “Mama, what is the matter?”, she said “Matron, my body has been feeling light since you told me to eat this and that, so for the past three days I have been eating cassava flour” then I told her “Mama but I have explained to you that this cassava flour … you have to reduce it because …. You are spoiling the work of the drug”. (health worker, Nigeria)

### Impact of formalised remote consulting

#### Strengths and benefits for patients, healthcare workers and primary healthcare facilities

Patients reported having confidence in their health worker when consulting by phone and appreciated efforts to follow them up. They felt cared for, supported and ‘not left alone’ (patient, Tanzania). Many found ‘no difference’ (patient, Nigeria) in the level of professionalism, communication and quality provided remotely and in-person.

It’s confidential and I trust them and also the treatment. They are very effective whenever I call them and they also call me back to check on me. I can’t keep anything from them. (patient, Nigeria)

Patients said that remote consultations saved them time, leaving ‘more time for my trade’ (patient, Tanzania) and other activities. Health workers echoed that patients saved time by not having to travel or wait in queues, including to ask simple questions such as where to go for a scheduled scan. Some patients said they preferred remote consulting because it was easier and more efficient to access busy health workers.

In both countries, health workers said that remote consultations improved their own, and the facility’s efficiency, enabling them to:

… go straight to the point; I think it is more efficient than even seeing patients physically. (health worker, Nigeria)

Solve small health issues [leaving…] time for other activities. (facility manager, Tanzania)

[Reach] a larger number of people. (facility manager, Nigeria).

Health workers identified improvements in the patient referral process. At one facility in Nigeria, for example, a health worker explained establishing a process of exchanging contact information with patients, setting up appointments for the patients at referral centres and providing them with special referral codes to present on arrival.

#### Challenges and strategies

Organising remote consulting required planning, resources, reminders and workarounds. For all health workers interviewed, the provision of airtime was identified as essential. Without funded airtime, health workers noted they would typically have to pay for calls themselves, sometimes because patients had insufficient airtime:

Even for the old woman who cannot [operate] the phone she would say “I borrowed the phone of one child…I don’t have money, help me, call back”. Then I will call the person back and we talk on the phone. (health worker, Nigeria)

In both countries, several patients said that they could not afford airtime and were thus unable to initiate a call without borrowing money or receiving funding. In a few cases, elderly patients did not have regular or easy access to a mobile device. In Tanzania, some patients and health workers reflected that, at an individual level, having a basic push phone (not a smartphone) reduced the range of remote services available, especially communicating/sending photos via WhatsApp. Network instability was also highlighted as a challenge in Tanzania, especially in remote rural villages or during heavy rains. Additionally, knowing which number to call—for patients and health workers—was challenging without established systems.

Participating in the trial required that facilities set up disease registers and record phone numbers for their open cohort of patients living with long-term conditions. Each facility approached this in a way that suited their working practices and many extended their system for patients beyond the trial. Some systematically recorded patients’ phone numbers during face-to-face treatment to facilitate follow-up, track their health progress or address challenges. Contact information for a close relative or friend was commonly collected for patients without phones. In Tanzania, some facilities designated specific times for staff to handle patient phone calls, with doctors assigned on different days. One facility followed up with patients biweekly to assess their condition and offer advice. Another facility advertised its phone number for patient inquiries from 06:00 to 19:00, encouraging calls for issues such as insulin dose adjustments. Some patients responded positively to this system, others were either unresponsive or unavailable.

When scheduling remote consultations, health workers noted that patients often required reminders. Where patients lacked access to a phone, both patients and health workers developed workarounds. For example, phoneless elderly patients would wait for a family member with a phone or borrow one specifically for the consultation. In Nigeria, one patient explained that her child would leave her phone when going out and taught her how to answer calls.

In both countries, to ensure that they were reachable for emergencies or planned consultations alike, health workers frequently gave out their personal phone numbers to patients and/or patients’ relatives. If a patient’s phone was unreachable, health workers described contacting relatives. One male health worker in Tanzania expressed concern that young women might be hesitant to share their phone numbers due to cultural sensitivities.

Health workers and patients occasionally initiated remote consultations at times inconvenient for the call recipient. In Nigeria, a health worker explained that a busy patient might respond to a call with, ‘I will call you back’, but then fail to do so. A patient recounted having to delay a consultation when travelling on a bus. In Tanzania, a health worker mentioned being unable to receive phone calls while consulting in-person and therefore deferring remote consultations until after the health worker’s working hours. When health workers received calls while outside of the facility, they found it challenging to document the consultation or provide prescriptions.

Some health workers encouraged patients to call at any time of day (or night), even when the health worker was off duty. This could intrude into their home life:

Then we usually tell [patients] to feel free to call us at any time of the day. In our families too we make them understand the people we are dealing with…. “Don’t be annoyed, I can receive any call at any time”. (health worker, Nigeria)

A health worker in Tanzania worried that taking calls or texting patients after hours, especially when using a personal phone, might raise unnecessary suspicion or cause conflict with a spouse/partner. Another explained that patients often called at ‘ungodly hours for a simple issue’, rather than in an emergency (Tanzania). In a case of misuse of remote consultation, a health worker in Nigeria mentioned blocking a patient’s number after they called repeatedly for minor concerns. This could hinder the success of remote consultations.

### Health system readiness for remote consultation

To understand health system readiness for remote consultation, we used the WHO Digital Investment Guide^6^ to analyse the ICT environment and broader enabling environment in the seven countries where we undertook stakeholder engagements. This guide is underpinned by principles of equity and universal health coverage.

#### ICT environment

Remote consulting requires sufficient mobile phone coverage. All study countries were experiencing a rapid increase in the proportion of the population with mobile cellular subscriptions, but where they were on this curve varied—in 2021, the national number of subscriptions per 100 people was 60 in Malawi, 66 in Uganda, 85 in Tanzania, 91 in Nigeria, 98 in Sierra Leone and over 100% in Ghana and Kenya,^19^ indicating substantial differences between countries. Alongside subscriptions, service coverage (ie, being in reach of a mobile broadband network) and usage are important indicators of connectivity.^23^ At the time of the study, sub-Saharan Africa had the widest gaps in usage (59%) and coverage (15%) globally, although rates varied by sub-regions, geographies and populations.^23^ *Local coverage* and *local ability to use available mobile services* are key for the local deployment of remote consulting.

#### Enabling environment

At the time, all study countries were advancing digital health and had national policies in place (online supplemental materials 4). All had deployed digital health tools, suggesting capacity in leadership, governance and multisector engagement. But stakeholders in Ghana, Tanzania and Sierra Leone had less experience. For all, changes in donor funding (shifting priorities and cutbacks) undermined the sustainability of digital tool implementation reliant on this funding channel.

Table 3 identifies health system enablers for upscaling remote consulting into existing primary care systems. Leadership and governance components were in place in each country. Supporting digital infrastructure such as e-prescriptions and online platforms for delivery of health worker training were available in a few countries. Training in remote consulting was only available in Nigeria and Tanzania (as REaCH training), and remote consulting was not yet in the scope of practice of healthcare workers in any. Provision of airtime for healthcare workers, service delivery guidelines and payment/billing systems for remote consulting had yet to be introduced. As a result of this work, we produced REaCH Service Delivery Guidelines (online supplemental materials 5) to introduce healthcare workers, managers and decision-makers to remote consulting. These are tailored for facility/local level use, with an emphasis on why, how and when to do remote consulting within the existing health system.

#### Informal remote consulting

Stakeholders from across the study settings recognised that informal remote consulting was already happening in their countries. They expressed concern about the burden placed on health workers and quality of care. They worried that health workers might be asked about health issues beyond their remit, would not know patients’ medical histories or have access to clinical records or would not know details of services relevant to patients/how patients might access them (eg, through referral). Across stakeholders, these concerns prompted discussion about needing policy-related changes (nationally) and planning (locally).

## Discussion

In our study settings, as elsewhere across sub-Saharan Africa, informal remote consulting was already happening.^11 14^ However, by participating in the REaCH trials, study facilities trained, planned for and formalised its delivery to some degree. REaCH training was well-aligned with the everyday work of facilities and health workers. It delivered aspects of talent, resources, alignment, implementation and nurture (TRAIN elements). Where available, trainees embraced WhatsApp for facilitator and peer support, although the requirement to engage synchronously outside of work hours caused some trainees difficulty. Facilities planned for remote consulting in ways adapted to their staffing schedules, appointment systems and lines of contact. Health workers used remote consulting to arrange patient follow-ups and patients phoned health workers to ask for advice about their treatment, find out about test results and seek prescriptions or referrals. Sometimes they phoned with urgent issues. Patients who had experience of remote consulting expressed confidence in the health worker they consulted. Patients and health workers felt remote consulting saved them time. Health workers perceived a reduction in work stress. However, it was not always straightforward or convenient for patients and health workers to contact each other remotely. Calls when off duty were usually accepted but could become burdensome for the health worker.

A large study in Ghana, Ethiopia and Malawi found enthusiasm among community health workers for informal remote consulting because it improved patient outcomes. However, they also felt burdened by having to pay for airtime and encroachment into their personal time.^11^ Our study found similar enthusiasm, which the training helped to harness towards formalising remote consulting into day-to-day primary care provision. What is not clear is how such formalisation interacts with health workers’ communitarian Ubuntu ethic,^24^ particularly for those living within the communities they serve where informal remote consultation may be expected of them. However, our training enabled facilities to plan for patient-centred care attending to equity and inclusion, as advocated by African Centres for Disease Control^25^ although workarounds were often needed and challenges remain. The training was cascaded to a range of cadres, including technical and administrative staff in Tanzania who were consulting with patients. The inclusion of such staff is an important consideration, given widespread task-shifting in sub-Saharan Africa.^26^

### Study strengths and limitations

The REaCH trials provided some of the organisational elements for delivery of the REaCH training including its cascade. The trials were designed to ensure these elements were scalable, except the expert facilitation. To rollout the trials rapidly in response to COVID-19, we had to accept this limitation. However, we have now identified from our process data key elements of facilitation and incorporated these into a standalone digital version of the training (available as an app on Google Play Store and Apple App Store, with links on the REaCH website: https://warwick.ac.uk/fac/sci/med/about/centres/wcfgh/research/remoteconsulting/reachtraining/). Although scalable in theory, REaCH training was not scalable in practice but we undertook stakeholder engagement to identify impediments to scale-up and to support training sustainability at local and national levels.

Patient participants were living with long-term health conditions and had consulted three or more times within the last year, demonstrating engaged care-seeking behaviour. For any consulting (face-to-face or remote) clinical assessment is required to determine the appropriate mode. Regardless of appropriateness, experiences of remote consulting might be different for both patients with limited health seeking interactions (face-to-face or remote) or acute health conditions; and the health workers they consult.

Data sources included documents produced without direct researcher involvement (WhatsApp transcripts, cascade reports, policies). These gave ‘unobtrusive’ (np) insights into the REaCH training process/policy context but we could not directly probe issues documented.^27^ Rather, we explored their insights with REaCH trainees and stakeholders, and triangulated and integrated our findings in analysis.^27^

In our interviews, virtually all of the trainees said that they liked the training. This may reflect social desirability bias.^28^ However, we have reports from patients and health workers of the training being used in practice.

### Recommendations towards sustainability and equity

At facility-level, the REaCH trials provided training materials, facilitators, IT support and airtime to support training and remote consulting. The training materials remain freely available for not-for-profit use (through the app and REaCH website). Stakeholders raised concerns about sustainability and scale-up beyond the resources provided by the trials. Interrelated action across the health system building blocks is needed at national and facility levels for remote consulting to become embedded in primary care delivery^3^ (as identified in table 3 for the study countries). Consistent with growing evidence in sub-Saharan Africa,^29–31^ our results indicate that access to remote consulting is shaped by poverty (unaffordability of airtime and phone access), locality, connectivity and digital infrastructure (limited/unstable in underserved rural areas, vulnerable to the weather) and potentially, the ‘smartness’ of available technology itself (eg, types of communication enabled by basic vs smartphones). Addressing these determinants requires coordinated action across sectors, including health, technology, communication and infrastructure. It also requires ongoing research into how different populations experience, adopt and adapt remote consulting. A better understanding of gendered experiences of digital health—both training and delivery—is similarly important for promoting equitable primary healthcare and building an inclusive health workforce.

## Conclusion

We have demonstrated successful training of primary care health workers for planning and embedding safe and trustworthy remote consulting, adapted to local needs for patients living with long-term conditions. The countries included in our stakeholder engagements have each started on at least some of the national level steps towards embedding remote consulting. For example, in Kenya, remote consulting was included in the national self-care guideline for reproductive health, which was approved by the Ministry of Health in June 2023. REaCH training is accredited for continuing professional development in Tanzania, Nigeria and Ghana. Meanwhile, informal remote consulting is likely to continue with its associated risks. With REaCH training, health workers can benefit from training and guidance on remote consulting. With collaboration between national regulatory bodies, facility managers, healthcare workers and communities, remote consulting can be piloted and then upscaled.

## Supplementary material

10.1136/bmjgh-2024-018780 online supplemental file 1

## Data Availability

Data are available upon reasonable request.
