# Supplementary material for: Formalising remote consulting in primary care services for underserved populations with long-term conditions in sub-Saharan Africa: a process evaluation and stakeholder engagement
Source: BMJ Glob Health. 2026 Oct 6;11(10):e018780. doi: 10.1136/bmjgh-2024-018780 (PMC13646971; doi:10.1136/bmjgh-2024-018780)
Supplement: Supplementary file 1 [file bmjgh-11-10-s001.pdf]

Formalising remote consulting in primary care services for underserved populations with long-term conditions in sub-Saharan Africa: a process evaluation and stakeholder engagement

Supplemental materials

1. **REaCH training: module learning outcomes**

Module title	Enabling Learning Outcome	Sub Enabling Learning Outcomes
1. Introduction: Why is remote consulting important?	Develop and strengthen motivation for engagement	Comprehend the importance of remote consulting
2. What digital devices and platforms can be used for mobile consulting?	Analyse different forms of digital communication in common use and consider how they might be used in health care	Identify forms of digital communication that currently exist
		Comprehend how the various forms of digital communications are commonly used and paid for
		Describe the changing patterns of use by the citizens
3. How does my role change and the care I provide my patients?	Discuss how digital communication changes the nature of the health professional and patient roles, and their interactions	Differentiate the various users of digital communication in health
		Describe the development, qualities and conduct of a digital health professional
		Describe the development, qualities and conduct of a digital patient
		Describe the development, qualities and conduct of a digital community
4. What new issues arise in remote consulting that are different to face to face care?	Summarise the enablers and barriers to implementing a digital communication service about clinical issues, including but not limited to: technical issues, communication skills, ethics, patient safety, cost, and sustainability	Analyse the change in roles for patients and health professionals involved in digital communication
		Describe the doctor – patient digital interaction process and its outcomes in various settings
		Demonstrate change in roles arising from digital communication for health professionals and patients
5. What patient outcomes can I expect?	Explain how digital communication with patients and between health professionals about clinical issues is	Describe the potential improvements in health outcomes arising from the use of digital communication

**Module title**	**Enabling Learning Outcome**	**Sub Enabling Learning Outcomes**
	likely to have benefit for patient care and health outcomes	Describe the benefits beyond the improvements of health outcomes of using digital communication for patients and health professionals
6 What is my plan for delivering my work remotely (and that of my team/colleagues)?	Develop a plan for a digital communication service about a health need identified from one's own practice, either with patients or between health professionals	Define a problem from clinical practice that can be addressed by digital communication
		Identify the opportunity for an intervention using digital communication to address the problem
		Describe the components of a digital communication plan
		Utilise a plan template to develop a realistic digital communication plan for patients or health professionals
		Describe how the plan could be monitored and evaluated.
7. What health behaviours will help or hinder the successful transition to remote consulting?	Summarise the enablers and barriers to implementing a digital communication service about clinical issues, including but not limited to: technical issues, communication skills, ethics, patient safety, cost, and sustainability	List the enablers and barriers for digital communication service for health issues<br><br>Explain how technical issues, communication skills, ethics, patient safety, cost and sustainability issues can hinder or facilitate digital communication in health
8. What qualities do you have and need to deliver remote healthcare and support your colleagues/teams	Reflect on the leadership qualities required to bring about change	Describe leadership qualities required to bring about change
		Utilise the clinical leadership framework to develop leadership skills in digital communication

2. Interview topic guides and coding structure used to guide the data analysis

Topic guide: District/facility manager and Tier 1 and 2 health workers

Please tell me about:

REaCH training

- your experience of ReaCH training (Tier 1)
- support from the facilitator and peers? (Tier 1)
- how confident did you feel to undertake cascade training (Tier 1)?
- your understanding of how your trainees planned to or have deployed remote healthcare (Tier 1)?

Organisation of care in your health facility

- how they have deployed remote healthcare?
- changes to your work pattern?
- changes to how the facility is organised?
- what do you think patients think and experience about remote consulting ?
- what is working well and why?
- what needs improving and why?
- what changes needed or made to enable remote consul ng (e.g. facility expenditure on airtime, phones, appointment system, guidelines, policy)?

Cascading

- What was your cascade training experience? (Tier 1 & 2)
- What topics did you prioritise in your cascade training? (Tier 1)
- What are the topics you most remember? (Tier 2)
- How have you been able to use the new knowledge and skills? (Tier 2)
- What has been your experience of informal discussion about remote consulting with your colleagues, patients, family and friends? (Tier 1 & 2)

Remote telephone consulting

What is your experience of remote telephone consulting? (Tier 1 & 2):

- Before the COVID pandemic
- During the COVID pandemic?
- Since the COVID pandemic?
- Since you undertook REaCH training/cascade training? (Tier 1 & 2)
- Please take me through what happened during your last remote consulta on (repeat for up to three remote consultations) (Tier 1 & 2)

If no experience of remote telephone consulting:

- Can you tell me about any plans to deliver care this way? (Tier 1 & 2)
- If you were to start, are there patients who may benefit first? (Tier 1 & 2)

Topic guide: REaCH facilitators

Please tell me about:

- your experience of ReaCH training modules
- What worked well or not in the training?
- What adaptations did you make to the training, why and what was the effect?
- What enables the Tier 1 trainees to cascade training successfully and what hinders this?
- How did the Tier 1 cascade training fit within other team activities?
- What have you learnt about identifying and recruiting the "best" Tier 1 trainees?
- Thinking about the TRAIN framework, can we talk about what your reflections on how the recruitment to, and delivery of, REaCH training and cascading could be strengthened to optimise the Talent/Resource/Alignment/Implementation and Nurturing?

Topic guide: Patients

Please tell me about:

- Your recent experience of talking to your health worker by phone?
- Can you take me through what happened during your last remote consultation?
- What impact did this have on you and how your managed your health?
- In what ways does remote consulting help you and what aspects are not so easy?
- How does availability of phone ownership impact on its usefulness?
- How does availability of type of phone impact on its usefulness?
- How does availability of airtime impact on its usefulness for accessing healthcare?
- What are the characteristics of any health consultation that make it feel safe and trustworthy?
- How has your experiences of remote consulting in the past year met up to your ideas of safe and trustworthy health care?

Coding structure used to guide the data analysis

1. **Facility Description**
2. **The training** (mechanisms of impact)
    - Overall reactions to the training
    - Training mediators
        - Facilitators/strengths
        - Barriers/challenges
    - Topics remembered/discussed
    - Cascading priorities
    - Cascading experiences
3. **Accounts of what the facility did in response to the training**
    - What was remote consulting like in the clinic prior to the intervention?
    - Preparation for remote consulting in the clinic
    - Preparation undertaken by patients
    - How was remote consulting implemented in this clinic?
    - What changed in the clinic because of the intervention?
4. **Remote consulting**

- General thinking about remote consulting
- Remote consulting mediators
    - Facilitators/strengths
    - Barriers/challenges

5. **Examples of actual remote consultations**
    - Reactions to remote consulting by people who experienced it
6. **Suggestions/recommendations**
    - For remote consulting training
    - For remote consulting
7. **Emerging issues/ideas** (new themes)

3. **Talent, resources, alignment, implementation and nurture (TRAIN)[1] in the delivery of REaCH training and its cascade in Nigeria and Tanzania**

Element	Levels	
	**Levels**<br>*Individual level:* REaCH Facilitators, Tier 1 and Tier 2 trainees<br>*Organisational level:* Primary Healthcare Facilities participating in the REaCH Trials<br>*Supra-organisational level:* National health and technology systems	
**Talent**	*Individual*	*Training enthusiasm, competence, communication and leadership skills.*<br>- Facilitators and Tier 1 trainees were perceived as friendly, approachable and available.<br>- Training was only undertaken in facilities with engaged managers who were committed to participating (REaCH inclusion criterion).<br>- REaCH facilitators were seen as experts in remote consulting training.<br>- Tier 1 trainers were viewed as proactive, motivating and knowledgeable
	Organisational	*Organisational processes for selecting facilitators, supporting training and developing trainers.*<br>- Facilitators were selected and briefed by REaCH trial team (2 per country).<br>- Tier 1 trainees were nominated by facility managers using study guidance on gender balance.<br>- There were no trial requirements for the selection of Tier 2 participants.<br>- Synchronous Whatsapp sessions were scheduled around working hours by facilitators.<br>- Facility managers made time for cascaded training to happen during the working day.
	Supra-Organisational	*Professional accreditation of REaCH training and inclusion of remote consulting in the scope of work*<br>- Training processes followed training norms for health workers in each country.<br>- REaCH training was not accredited for continuing professional development at the time of the trials (both countries).<br>- Remote consulting was not included in the scope of practice of healthcare workers (nationally in both countries).<br>- We heard several examples of informal remote consulting happening due to COVID-19 and where community members and health workers were socially connected.
**Resources**	*Individual*	*Financial and physical resources for doing the training and training others*<br>- It was a training requirement that Tier 1 trainees had smartphone access to access the Moodle app.<br>- Most Tier 2 trainees had smartphones although some had feature phones.<br>- In Nigeria, Tier 1 trainees used WhatsApp rather than the Moodle App. In Tanzania Tier 1 trainees used both platforms.<br>- In Nigeria, all face-to-face Tier 2 training was supported by Whatsapp groups, compared to 9/20 training groups in Tanzania (due to limited internet connectivity and lack of access to WhatsApp).
	Organisational	*Capacity and willingness by facility to release staff to train and be trained*<br>- Facilities were willing to engage with the training (pre-requisite for study participation).<br>- Facility managers were supportive and involved, with many themselves doing the training.<br>- REaCH trials paid the facilitators and provided each facility with airtime.<br>- REaCH team provided IT support. Several trainees were unfamiliar with the training platforms and needed technical support.
	Supra-Organisational	*Mobile phone network coverage, access/usage of WhatsApp and other social media*<br>- Network instability was a challenge<br>- At the time of writing: WhatsApp is the dominant social media platform in Nigeria. In Tanzania, Tik Tok and WhatsApp have similar user numbers

**Alignment**	*Individuals*	*Alignment of training with individual's work*<br>- Most trainees felt the training addressed their anxieties about doing remote consulting
	Organisational	*Alignment of training with facility priorities*<br>- Facility managers said REaCH training complemented facility priorities.<br>- Training on planning for remote consulting was valued and addressed trainees' worries about remote consulting
	Supra-Organisational	*Published health policies*<br>- National telehealth strategies were in place.<br>- Several trainees questioned whether training for, and implementing, remote consulting was realistic and sustainable without basic infrastructure and resources.
**Implementation**	*Individuals*	*Evidence for training skills gained and training developed and delivered.*<br>- Tier 1 trainees said they had acquired skills and felt confident training colleagues.<br>- All Tier 1 trainees cascaded the training within their facilities.<br>- Tier 2 trainees generally reported good experiences of training.<br>- Tier 1 trainers used several training methods from formal slide presentations at large group gatherings to one-to-one updates or less formal group discussions (once-off or regular meetings), mostly with small groups, but, in one facility, involving 16 colleagues.<br>- Facilitators scheduled *synchronous* WhatsApp sessions mainly in the evenings, to reduce working day disruption.
	Organisational	*Organisational strategy to cascade the training*<br>- All facilities developed strategies for cascading the training.<br>- Tier 1 trainees were expected to join a WhatsApp group discussion, often after working hours
	Supra-Organisational	*Strategy to expand training*<br>- The process to accredit REaCH training with professional bodies was started (Nigeria) and continued in Tanzania during the study.
**Nurture**	*Individuals*	*Teaching of "soft" skills*<br>- Facilitated sessions provided trainees with opportunities for discussion, clarification and problem-solving.<br>- Facilitators were seen to model a democratic, inclusive approach, enabling the cascading of a similar training style and ethos.<br>- Most trainees anticipated technical challenges with doing remote consulting, and many of these challenges were experienced – and solved – in training. Solutions included ensuring that all participants had connectivity, airtime/data and knew how to use the technology.
	Organisational	*Trainer mentoring*<br>- Tier 1 trainees asked facilitators for support with planning and running their cascaded training.
	Supra-Organisational	*Making available learning resources*<br>- REaCH training is available free to use (not for profit).<br>- South-South transfer of knowledge and learning.

The levels of impact summarise TRAIN framework elements relevant to REaCH training and its cascade. Text applies to both Nigeria and Tanzania unless specified.

4. Global and national policies guiding the embedding of remote consulting within primary care

World Health Organization (WHO)	Regional	National policies and initiatives in the study countries
Recommendations on digital interventions for health system strengthening (2018): supports the use of telemedicine to bolster coverage and quality of services for both client-to-provider telemedicine and provider-to-provider telemedicine.<br><br>WHO Global Strategy on Digital Health 2020-2025U (2020): Principles for addressing barriers and implementing digital health at scale include:<br>• Take a systems approach and mainstream digital health and virtual care<br>• Establish an intersectoral governance system<br>• Create national agencies and policies to plan, implement and monitor<br>• Protect and strengthen privacy and data<br>• Ensure local relevance and cost-effectiveness<br>• Monitor and evaluate consistently<br>• Fund digital health as part of integrated health service delivery<br>• Collaborate and recognise role of private sector<br><br>Digital Implementation Investment Guide (DIIG) (2020) and DIGG: Quick Deployment Guide (2022): Step-by-step guidance for planning, costing and implementing digital health interventions within a digital health enterprise.<br><br>Consolidated telemedicine implementation guide (2022): Brings together key telemedicine resources and evidence and uses DIIG framework for planning, costing	**African Union:** Digital Transformation Strategy for Africa, 2020-2030: Emphasizes regional integration as part of creating a pan-African Digital Single Market which, at a health level, aims to 'connect all of Africa's health facilities and workforce by 2030, advancing health data protection, portability, interoperability, and governance…' through the Africa CDC and Smart Africa Initiative.<br><br>**Africa CDC:** Digital Transformation Strategy, March 2023 (v1): Guiding principles for strengthening inclusive digital health systems on the continent include patient-centredness, open standards and DPIs/DPGs, regional integration of capabilities, reuse and improve, design for scale and build for sustainability, digital leapfrog and innovative solutions, workforce empowerment.<br><br>**East African Community (EAC):** The Digital REACH initiative (2017) (with roadmap and strategy: 2019-2028): Emphasizes regional integration and cross-border health.<br><br>**Economic Community of West African States (ECOWAS):** Vision 2050 (2022): Digitalisation is a cross-cutting theme, including for health, requiring 'the establishment of appropriate regulatory frameworks, sector-adapted governance, as well as affordable, secure and quality digital infrastructure'.	**Ghana**<br>National eHealth strategy (2010)<br><br>**Kenya**<br>Kenya National eHealth policy 2016-2030: Remote consultation is legal and encouraged under Kenya National eHealth policy<br>Telemedicine Guidelines and the Kenya Data Governance Framework currently under development by Department of Health.<br><br>**Malawi**<br>Malawi National Digital Health Strategy 2020-2025: Digital technologies are key for UHC. Telemedicine has been successfully piloted.<br>Monitoring, Evaluation, and Health Information Systems Strategy 2017-2022<br>Malawi National Community Health Strategy 2017-2022: 2022 target: 50% community health teams to be using mHealth for integrated service delivery, data collection, and supervision.<br><br>**Nigeria**<br>National Health ICT Strategic Framework 2015 (eHealth Strategy) guides the alignment of technology investments in the health system. Regulated remote consulting could fit within an updated framework.<br><br>**Sierra Leone**<br>Sierra Leone eHealth Strategy of 2018<br>National Digital Health Strategy (2018 – 2023): This strategy includes telemedicine and other remote access systems to ease patient interaction, especially in remote settings.<br><br>**Tanzania**<br>Tanzania National Digital Health Strategy (2019 – 2024)<br>Remote consultations are implemented in Tanzania<br><br>**Uganda**<br>National eHealth Policy (2016)<br>National eHealth Strategy (2017-2021): Telehealth/medicine is a strategic objective.

and implementing interventions.<br>Broadband Commission Working Group report on The Future of Virtual Health and Care (chaired by WHO and Novartis) (2022): Proposes a roadmap/policy maturity framework with policy steps necessary to ensure virtual health and care helps countries address health equity and access challenges.	**Southern African Development Community (SADC):** Vision 2050: Emphasizes health/healthy lives through 'strengthened and harmonised regional health systems for the provision of standardised and accessible health services to all citizens and addressing threats caused by health pandemics' (2020).<br>2021 SADC Regional Indicative Strategic Development Plan (RISDP) 2020-2030: Seeks to build 'improved, accessible, and responsive regional health systems'.	Community Health System Strategy launched 2023: Community Health Approach as vehicle for REaCH

5. REaCH Service Delivery Guidelines for remote consulting

Scope of Guidelines
These guidelines are for use by those planning, implementing, and undertaking service delivery for patients in primary and ambulatory care. For successful remote consulting there are health system issues that need attention:

- Provision of airtime for remote consulting
- Payment for remote consulting
- How patients obtain prescriptions and medication
- Arrangements for examination of patients who need it
- Access to tests and referrals.

What is Remote consulting?
Remote consulting is when the healthcare provider and patient are not face to face and communication is via a digital or remote communication device such as mobile phone. if using a mobile phone, this can be of any type for remote consulting. Features such as camera and access to data allows for additions to basic remote consulting such as transmission of photographs and text-based information. Remote consulting is also known as mobile consulting, mconsulting, telephone consulting, teleconsulting, digital consulting.

In a remote consultation, a healthcare worker uses all the knowledge, skills and experience they already possess and would ordinarily use in a face-to-face consultation.

This guideline does not prescribe who provides the healthcare, how or for what purpose. However, research evidence indicates that remote consulting is effective, safe and trustworthy when undertaken by health workers at a facility with people already known to the health facility, for example those needing follow up for long term conditions or after initiating treatment[1,2].

What type of consultation can be undertaken remotely?
Remote consultations compliment facility visits when the patient sees the healthcare worker face-to-face. They can be used for:
- planned care, such as reviewing patients after treatment or as part of managing a long-term condition
- responsive care when a patient initiates the contact.

What makes a remote consultation safe and trustworthy?
Remote consultations are safe and trustworthy when delivered by a health worker trained in remote consultation[2].

Timely access to a known health worker can increase trust between the patient and the healthcare provider[3]. Remote consultation allows this timely access.

For each remote consultation, the health worker assesses whether it is appropriate or whether face-to-face consultation is needed. For example, for physical examination or if the patient struggles to communicate by phone.

There are remote examinations that may be sufficient for safe clinical practice, for example, a patient sends a photo of their condition (e.g., a rash) or their blood pressure readings.

How do health workers conduct remote consultations?
Remote consultations involve a healthcare worker and a patient. Another person such as a family member, advocate or interpreter may be present with permission of the patient.

The healthcare worker uses all their existing knowledge, skills and experience within the consultation. They listen carefully to the patient just as they would face-to-face but verbally indicate that they are actively listening. An indication of such active listening is to ask the patient clarifying questions about what the patient has just told them. The healthcare worker needs to be alert to what they may be missing because they cannot see the patient and ask questions that help fill these gaps. If questions are insufficient to fill gaps, then they consider the use of photos or see the patient face-to-face.

The healthcare worker undertakes the remote consultation where and when they can ensure communication is technically satisfactory via the phone – they can hear the patient and the patient can hear them. It may be important to know the type of phone being used by the patient, so they know whether it is possible for the patient to send a photo or receive written health information. The healthcare worker needs to ensure they:

- Cannot be overheard or recorded,
- Have access to the patient's clinical records for continuity of care,
- Are satisfied as to the identity of the person they are talking to,
- Have asked the patient about confidentiality and privacy of the patient side of the consultation and that they are comfortable to continue.

If the healthcare worker sends a text message, they need to ensure the

- patient can receive text messages on their phone,
- text message will not be visible to other people without patient consent,
- patient can read a text message (consider eyesight and literacy and local language),
- text message is written clearly with no typing or grammatical errors, using wording accessible by lay people but avoiding informality[3].

Healthcare providers record in patient clinical notes for remote consultations and text messages in the same way they would record a face-to-face consultation.

How do healthcare facilities plan for remote consultation?
Healthcare workers need training on how to conduct remote consulting. This training is not about the clinical content of their consultation but how to plan and deliver a safe consultation remotely[4]. The following are issues to consider when planning remote consulting for a healthcare facility.

What technical arrangements are needed for remote consulting?
Remote consultations require mobile phones, airtime, mobile signal, and mobile numbers. To contact patients, healthcare facilities need a record of each patient's mobile number. For patients without their own phone, it will be necessary to record the mobile number of a family member/friend/neighbour. Health facilities need a phone number for patients to call and from which health workers call patients. Telecommunication companies can provide mobile phone arrangements suitable for health facilities.

What do health workers need to know about the integration of remote consultation with the health system locally?
Health workers need to be aware of how remote consultation is integrated with the local health system including provision of medication, payment of fees, arrangements for physical examination, access to diagnostic tests and referrals for treatment.

How does a facility triage and schedule remote consultations safely?

As with face-to-face consultations, for clinical safety some patients need attention urgently and others can wait for a scheduled appointment. Facilities need to plan how to triage patients, so the patient speaks to the right health worker with the appropriate level of urgency. They need to plan an appointment system for scheduling remote consultations for less urgent health concerns. To maintain the boundary for health-workers between work time and personal time, remote consultations should be scheduled within the normal work time of the health worker.

How do patients plan for remote consultation?
Patients should be encouraged to plan to receive a scheduled call from a health worker. They need to ensure they have with them the phone with the phone number used by the clinic and that it is charged, and that they are in a place with mobile signal and unlikely to be undisturbed. If a health worker makes an unscheduled call to a patient, they should ask the patient if they are able to take the call and offer to phone back when the patient can plan to receive the call.

How does the facility tailor the provision of remote consulting to the needs of the community they serve?
If a community is unfamiliar with the idea of remote consulting, consider undertaking community engagement activities.

Ensure arrangements for remote consultation are made clear to patients. This may include advertising to all patients or on a patient-by-patient basis depending on the service being delivered remotely. Patients need to know when it available, how to access it, who will respond and the timescale for response and what it provides.

Consider which patient groups are most likely to benefit from remote consulting and plan implementation accordingly. These patient groups may include those living at a distance from the health facility, working people who will lose income if they attend the facility for their ongoing health problem, those who struggle to pay for transport to the facility.

Consider which patient groups are likely to struggle to access remote consulting. Plan alternative arrangements for these groups for example, continuing to consult face-to-face or arranging for a family member or community health worker to assist individuals with remote consulting.

References
1. Griffiths F, Watkins JA, Huxley C, Harris B, Cave J, Pemba S, et al. Mobile consulting (mConsulting) and its potential for providing access to quality healthcare for populations living in low-resource settings of low-and middle-income countries. Digital Health. 2020;6:2055207620919594.
2. Sturt J, Omigbodun A, Pemba S, Griffiths F. Determining Trustworthiness & Safety of Remote Consulting in Primary Healthcare for Chronic Disease Populations in Africa 2020 [Available from: https://www.kcl.ac.uk/research/reach-trial.
3. Griffiths F, Bryce C, Cave J, Dritsaki M, Fraser J, Hamilton K, et al. Timely digital patient-clinician communication in specialist clinical services for young people: a mixed-methods study (the LYNC study). Journal of Medical Internet Research. 2017;19(4).
4. Downie A, Mashanya T, Chipwaza B, Griffiths F, Harris B, Kalolo A, et al. Remote Primary Healthcare Consulting in Low- and Middle-Income Countries: Feasibility Study of an Online Training Program (REaCH) to Support Care Delivery During the COVID-19 Pandemic. JMIR Formative Research. 2022;6(6):e32964.

6. BMJ Global Health Author Reflexivity Statement

Adapted from Morton, B., Vercueil, A., Masekela, R., Heinz, E., Reimer, L., Saleh, S., Kalinga, C., Seekles, M., Biccard, B., Chakaya, J., Abimbola, S., Obasi, A. and Oriyo, N. (2022), Consensus statement on measures to promote equitable authorship in the publication of research from international partnerships. Anaesthesia, 77: 264-276. https://doi.org/10.1111/anae.15597

**Study conceptualisation**	
1. How does this study address local research and policy priorities?	Improving access to healthcare for underserved communities is a global concern, including in sub-Saharan Africa. Remote consulting can facilitate access by bridging key barriers of geography, transport and costs. A form of telemedicine, it is a strategic priority for the African Union and national and local decision makers on the continent.<br><br>Working at the local level, in primary healthcare facilities in Nigeria and Tanzania, our study examines the process of training health workers to plan for and implement formal remote consulting (REaCH) in their own facilities. We also explore health system challenges for scaling-up formal remote consulting in primary care in sub-Saharan Africa.<br><br>In preparation, we consulted with local and national decision-makers, health facility managers, frontline health workers, and community leaders and representatives at each study site, to establish their key priorities. We piloted the REaCH training in Tanzania and made adjustments. Frontline healthcare workers and facility managers received REaCH training and, together with community members with long-term healthcare

	conditions, were included as study participants.
2. How were local researchers involved in study design?	Local researchers, working at the University of Ibadan and SFUCHAS, Tanzania make up over half of our authorship team (n=7/13). Project PIs and leads from each country were actively involved in developing the proposal. Once funded, our group met regularly (every 2-4 weeks for the project duration) to first design the study, develop the tools and seek ethical approval, then implement the data collection and undertake the analysis and write-up.<br><br>A similar mix of researchers from LMICs (15/26) and the UK (11/26), was involved in implementing and analysing the REaCH Trials as part of our Collaboration group.
**Research management**	
3. How has funding been used to support the local research team(s)?	Working in line with funding and institutional conditions, we sought to equitably distribute the budget between all research partners, with more than half of the salary budget allocated to locally based research teams (in LMICs), along with most of the research activity expenses. Funding was used to support salaries, overheads and field costs, as guided by our collaboration agreement and funding terms.
**Data acquisition and analysis**	
4. How are research staff who conducted data collection acknowledged?	Research staff who conducted data collection are acknowledged as follows:<br><br>*In the contributions:*<br><br>Within the author group: TM, OF led the data collection in Tanzania and Nigeria.

	TM, ADA, AI, AA collected data in the study sites.<br><br>The REaCH Trials Collaborative Group: BC, TM, MA, and OF led the data collection. EA, JOA, SIW, BH, and OF directly accessed and verified the underlying data.<br><br>*Acknowledgements:* We acknowledge Marcusy Balagaju, Frola Theophil Stephano in Tanzania, and Abiola Oladejo, Omolara Popoola, Habeeb Alabi, Oluwatoyin Oluyinka, Precious Ayodele and Rebecca Ojuade in Nigeria for their important contributions to data collection during the REaCH trials.
5. How have members of the research partnership been provided with access to study data?	All data were electronically transferred from local secure storage in both countries to a secure storage area hosted by the Clinical Trial Unit at the College of Medicine, University of Ibadan. Research teams from Nigeria, Tanzania and UK had access to the data for data entry, quality monitoring, and analysis.
6. How were data used to develop analytical skills within the partnership?	Team members worked together in small groups (within and across countries), to analyse different data sources and build themes. We discussed our codes, themes and findings throughout the process, using our regular meetings to demonstrate theme-building and explore differences/similarities in interpretation.
**Data interpretation**	
7. How have research partners collaborated in interpreting study data?	All research partners collaborated in interpreting the study data. We held regular meetings and worked collectively (using meeting discussions, writing, shared documents) to interpret the data and test and refine our collective understanding.
**Drafting and revising for intellectual content**	

8. How were research partners supported to develop writing skills?	In extension of the data analysis process, team members worked together in small groups (within and across countries) to write up findings from the different data sources. We met regularly and used tracked changes and comments to draft and redraft our writing. All authors contributed to the development and write-up of our manuscript.
9. How will research products be shared to address local needs?	Our manuscript will be published as open access. Research partners in each country will disseminate the research findings using our extensive institutional networks. Senior country leads will work with research teams and university media officers to disseminate the results on the ground. We are aiming to write up our findings for a publicly accessible publication such as *The Conversation Africa*.
**Authorship**	
10. How is the leadership, contribution and ownership of this work by LMIC researchers recognised within the authorship?	All LMIC researchers who worked on this study (7/13 researchers) are recognised in the authorship team, for their roles as leaders (e.g. in the role of joint-first author and senior country leads) and contributors as per the authorship criteria. These contributions are identified in our contributorship statement and in the manuscript's methods text (which identifies specific research activities undertaken by individual authors).
11. How have early career researchers across the partnership been included within the authorship team?	Just over half of the members of the authorship team (7/13) are considered early-career researchers (n=4 in LMICs, 3 in UK), with two mid-career (n=1 in LMIC, 1 in UK) and four at senior level (n=2 in LMICs, 2 in UK).<br><br>A similar mix of career stages is reflected in the wider REaCH collaboration.

12. How has gender balance been addressed within the authorship?	Almost two thirds (n=8/13) of the authorship team are women, with three leading the study and writing process (as joint-first and senior authors).
**Training**	
13. How has the project contributed to training of LMIC researchers?	All researchers benefitted from training in research methods and study write-up, with scheduled training sessions and through participation in regular team meetings (which provided ad hoc training and grounded learning). Training was delivered by team members with contextual and methodological expertise, from both LMICs and the UK, across the research cycle, including in obtaining ethical clearance, study design, data collection and analysis.
**Infrastructure**	
14. How has the project contributed to improvements in local infrastructure?	The project has contributed research skills (methods, data collection, analysis, write-up) to all researchers involved. Most of the study authors have continued to undertake research within their institutions or have gone on to further research-related employment or studies, thereby contributing to local capacity in digital health and research.
**Governance**	
15. What safeguarding procedures were used to protect local study participants and researchers?	Across both study sites, to protect local study participants and researchers, we followed existing institutional safeguarding guidelines and procedures (as ethically approved). Field teams received training in the ethics and safety procedures of conducting research and we used our regular meetings to address any issues or challenges that arose.
